# Eye-Visible Oxygen Sensing via In-Situ Synthesizing Blue-Emitting Cu(I) Cluster in Red-Emitting COF: Characterization and Performance

**DOI:** 10.3390/ma15134525

**Published:** 2022-06-27

**Authors:** Peibin Zhu, Lixiong Lin, Wen Chen, Liang Liu

**Affiliations:** 1 School of Ocean Information Engineering, Jimei University, Xiamen 361021, China; peibin.zhu79@outlook.com (P.Z.); chenw886@163.com (W.C.); 2 School of Materials Science & Engineering, Jiangsu University, Zhenjiang 212013, China; reserch419@outlook.com

**Keywords:** COF, micropores, oxygen-sensing, in-situ synthesis, halogen-bridged cluster

## Abstract

Covalent organic frameworks (COFs) have shown virtues of well-defined and uniform pores with structural diversity, including the shape, size and even chemical nature of pores. These features are excellent for the application of O_2_ gas optical sensors. In this paper, two oxygen probes based on halogen-bridged Cu cluster were in-situ synthesized in the micropores of COFs, to allow a uniform distribution. The resulting composite samples were characterized in detail to confirm the successful probe loading. The doping level was determined as ~22%. The halogen-bridged Cu clusters showed blue emission peaking at ~440 nm, while COF host showed red emission peaking at 630 nm. These halogen-bridged Cu clusters had long emissive lifetime of ~6.7 μs and high emission quantum yield of 0.30 in pure N_2_ atmosphere. Given pure O_2_ atmosphere, lifetime and quantum yield were quenched to 2.5 μs and 0.11, showing oxygen-sensing possibility. A linear oxygen-sensing calibration curve was observed, with sensitivity of 12.25, response time of 13 s and recovery time of 38 s. Sample emission color was changed from blue to red when testing atmosphere was changed from pure N_2_ to pure O_2_, which was detectable by eyes.

## 1. Introduction

Porous materials are a class of important host to support functional component in the fields of catalysis, optoelectronics, drug storage/transportation and sensors, which makes the development for porous materials always an attractive topic [1,2,3,4]. As a class of attractive porous crystalline polymers, covalent organic frameworks (COFs) have shown virtues of well-defined and uniform pores [5,6,7]. Their organic building components allow structural diversity, including the shape, size and even chemical nature of pores [8]. These features are excellent for the application of O_2_ gas optical sensors. It is well known that O_2_ is an important life-supporting gas and its quantification is always an important task in the field of medical treatment, industry, manufacturing and food preservation.

To construct an O_2_ gas optical sensor with linear sensing response, photosensitizers should be uniformly loaded into these COF pores, so that the microenvironment around each photosensitizer molecule is the exactly the same to the others [9,10]. Zhang and coworkers have demonstrated a doping method based on ionic exchange for MOF (metal-organic-framework) materials with improved linearity of calibration curves [11,12]. However, the backbone of COF materials is usually neutral, which denies the possibility of dopant loading via an ionic exchange reaction. Some alterative doping methods make the dopant loading generally an inhomogeneous one, leading to a down-bending calibration curve, compromising sensing linearity and sensitivity [13,14]. It is thus a challenge to realize a uniform dopant loading in COF materials.

In addition to the above mentioned two-step loading method: first dopant synthesis and second dopant loading (into COF), there is still an alternative way to realize dopant loading in COF materials, which is the in-situ dopant synthesis in COF. The in-situ synthesis/doping definitely avoids the limitations for COFs and dopants, so that uniform dopant distribution in COF matrix can be ensured, without phase separation or local aggregation [15,16]. On the other hand, the in-situ dopant synthesis can lay down some rules in front of probes, such as moderate synthetic procedure to preserve COF structure, not too acidic and not too alkaline condition to decompose COF structure, no interaction with COF components, along with size matching for COF pores. In addition, there are still general requirements for an oxygen-sensing probe, including long-lived excited state to allow O_2_ collision/quenching, photostability to ensure signal stability and high emission yield to give strong enough signal [9,10].

It seems that halogen-bridged Cu cluster with lifetime of microseconds and emission yields around 0.3–0.8 can satisfy the above requirements. Most halogen-bridged Cu clusters can be easily synthesized at ambient condition by mixing Cu(I) compounds with proper ligands. Volz and Brase have mentioned that their emission is quenchable by O_2_, which confirms their potential of being an O_2_ probe [17]. Guided by the above consideration, in this work, we firstly synthesized two halogen-containing COF hosts (denoted as Br-COF and Cl-COF), then halogen-bridged Cu(I) cluster was in-situ synthesized in each pore of Br-COF/Cl-COF. The resulting composite sample, denoted as CuPX-COF (X = Br or Cl), was firstly characterized and then explored for oxygen-sensing. Corresponding parameters of the halogen-bridged Cu(I) cluster, denoted as CuPX (X = Br or Cl), were recorded and discussed for comparison.

## 2. Experimental Details

### 2.1. Reagents and Apparatus

**Scheme 1:**
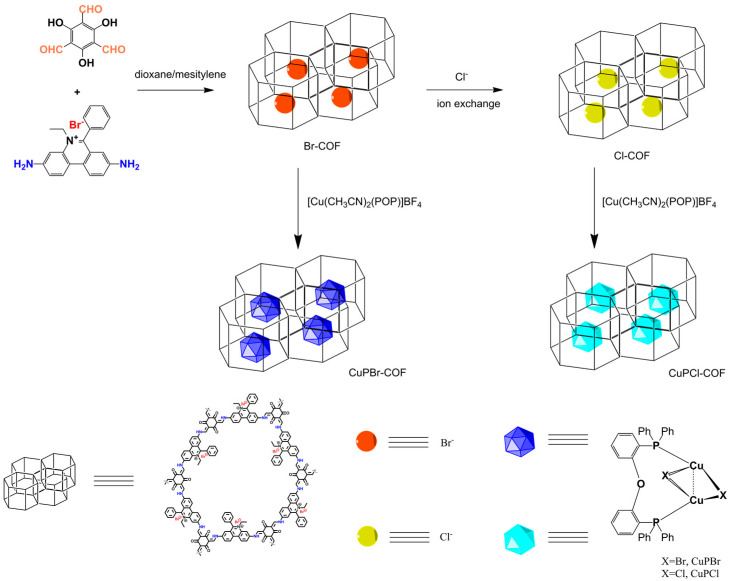
A synthetic strategy for CuPX-COF, X = Br and Cl.

A schematic presentation for the synthesis of this work is shown as Scheme 1. All chemical reagents used in this work were AR (analytical grade) grade ones and used as received, including Cu(BF_4_)_2_, bis [2-(diphenylphosphino)phenyl]ether (POP), 3,8-diamino-5-ethyl-6-phenylphenanthridin-5-ium bromide, 2,4,6-trihydroxybenzene-1,3,5-tricarbaldehyde, phloroglucinol, hexamethylenetetramine, trifluoroacetic acid and p-toluenesulfonic acid. Sample characterization was finished by below methods and apparatuses. Powder and single XRD data were collected on a D/MAX2550 (Rigaku, Tokyo, Japan) diffractometer (1.54 Å) and a Bruker SMART APEX II crystal diffractometer (Mo Kα radiation, Karlsruhe, Germany). NMR spectra were recorded by an Avance III 400 WB spectrometer (Bruker, 100.62/300 MHz, 9.39 T, Karlsruhe, Germany). Elemental analysis was performed by a Carlo Erba 1106 elemental analyzer (Carlo Erba, Milan, Italy). Emission spectra were recorded by an F7000 (Hitachi, Tokyo, Japan) spectrometer. SEM (scanning electron microscopy) and TEM (transmission electron microscopy) images were obtained on an S-4800 microscope (Hitachi, Tokyo, Japan) and a JEM-2010 microscope (JEOL, Tokyo, Japan). Microporous parameters were determined by a Quantachrome autosorb iQ_2_ analyzer (Quantachrome Instruments, Boynton Beach, FL, USA). MS spectra were obtained on an Agilent 1956B LC/MS spectrometer (Agilent, Santa Clara, CA, USA). N_2_ adsorption/desorption was performed by a Quantachrome autosorb iQ_2_ analyzer at 77 K (liquid nitrogen, Quantachrome Instruments, USA). Thermogravimetric analysis (TGA) was performed on a Perkin–Elmer thermal analyzer (PerkinElmer Analyzers, USA). Time dependent density functional theory (TD-DFT) calculation was performed on CuPBr at RB3LYP/LANL2DZ level, using its single crystal structure as initial geometry.

### 2.2. Synthesis of Br-COF and Cl-COF

Br-COF was synthesized following below method [15]. Firstly, a mixture of hexamethylenetetramine (54 mmol), phloroglucinol (24 mmol) and trifluoroacetic acid (40 mL) was stirred at 100 °C for 7 days in N_2_ atmosphere. After cooling, H_2_O and CH_2_Cl_2_ were added (100 mL + 100 mL). Organic phase was extracted and vaporized to give 2,4,6-trihydroxybenzene-1,3,5-tricarbaldehyde. Yield: 12%. Anal. Calcd. For C_9_H_9_O_6_: C, 51.44; H, 2.88; N, 0.0. Found: C, 51.26; H, 2.96; N, 0.04. ^13^C NMR (CDCl_3_) δ 196.2, 172.6, 103.2.

The as-synthesized 2,4,6-trihydroxybenzene-1,3,5-tricarbaldehyde was mixed with dioxane (5 mL), mesitylene (5 mL) and aqueous acetic acid (1 mL, 6 M), then 3,8-diamino-5-ethyl-6-phenylphenanthridin-5-ium bromide (1.5 mmol) was added. The resulting mixture was frozen at 77 K, degassed and then sealed in a pyrex tube. After being heated at 120 °C for 3 days, crude product was collected, refluxed by tetrahydrofuran/ethanol for 24 h, dried at 100 °C overnight to yield Br-COF as deep red powder. Yield: 70%. Anal. Calcd. For C_162_H_120_N_18_O_12_Br_6_: C, 65.07; H, 4.04; N, 8.43. Found: C, 65.27; H, 4.21; N, 8.25. ^13^C NMR (solid) δ 190.5, 167.1, 150.3, 144.7, 135.9, 130.4, 127.8, 113.6, 47.3, 18.4.

Cl-COF was synthesized following below ionic exchange method. The as-synthesized Br-COF (2.0 g) and NaCl (5 g, excess) was dispersed in H_2_O/methanol (1:1, 20 mL). After being stirred for 24 h, solid product was collected and washed with plenty of water. The above ionic exchange procedure was repeated for four more times. Finally, the solid product was washed with water and dried at 100 °C overnight give Cl-COF as deep red powder. Yield: 90%. Anal. Calcd. For C_162_H_120_N_18_O_12_Cl_6_: C, 71.44; H, 4.44; N, 9.26. Found: C, 71.27; H, 4.62; N, 9.11.

### 2.3. Synthesis of CuPBr-COF and CuPCl-COF

[Cu(CH_3_CN)_4_]BF_4_ was firstly prepared by refluxing a mixture of Cu(BF_4_)_2_ (100 mmol) and Cu powder (120 mmol) in CH_3_CN (50 mL) [18]. [Cu(CH_3_CN)_4_]BF_4_ (10 mmol) and POP (5 mmol) were mixed in DMF (10 mL) to form [Cu(CH_3_CN)_2_(POP)]BF_4_ solution. The above obtained Br-COF (0.5 g) was dispersed in ethanol (20 mL) and stirred for 30 min. Then the two solutions were mixed together and stirred for 8 h under ambient condition. The solid product was collected and washed with DMF and ethanol sequentially. After being dried in vacuum at 50 °C overnight, CuPBr-COF was obtained as deep red powder. Anal. Calcd. For C_162_H_120_N_18_O_12_Br_4_:C_36_H_28_Br_2_Cu_2_OP_2_: C, 65.05; H, 4.08; N, 6.90. Found: C, 65.00; H, 4.08; N, 6.92. Their composition was further discussed by thermal gravimetric analaysis which was discussed below.

CuPCl-COF was synthesized following a similar procedure, except that Br-COF was replaced by Cl-COF in this run. Anal. Calcd. For C_162_H_120_N_18_O_12_Cl_4_:C_36_H_28_Cl_2_Cu_2_OP_2_: C, 70.17; H, 4.40; N, 7.44. Found: C, 69.58; H, 4.25; N, 7.29. Their composition was further discussed by thermal gravimetric analaysis which was discussed below.

### 2.4. Synthesis of Reference Compounds CuPBr and CuPCl

Two reference compounds, CuPBr and CuPCl, were synthesized following below procedure. [Cu(CH_3_CN)_4_]BF_4_ (10 mmol) and POP (5 mmol) were mixed in DMF (10 mL) to form [Cu(CH_3_CN)_2_(POP)]BF_4_ solution. Then 12 mmol of cetyltrimethylammonium bromide or hexadecyltrimethylammonium chloride was added and stirred for 90 min. Crude product was washed with ethanol. CuPBr yield: 63%. ^1^H NMR (DMSO-*d*_6_): δ 7.36–7.31 (m, 18H), 7.18–7.13 (m, 8H), 6.82 (s, 2H). ^31^P NMR (DMSO-*d*_6_): δ 1.4 (brs, *W*_1/2_ = 325 Hz). MS calculated for C_36_H_28_Br_2_Cu_2_OP_2_: 825.45, found: *m*/*z* 823.9 [M-H]^+^. Anal. Calcd. For C_36_H_28_Br_2_Cu_2_OP_2_: C, 52.38; H, 3.42; N, 0.00. Found: C, 52.31; H, 3.51; N, 0.06.

CuPCl yield: 60%. ^1^H NMR (DMSO-*d*_6_): δ 7.36–7.31 (m, 18H), 7.18–7.12 (m, 8H), 6.82 (s, 2H). ^31^P NMR (DMSO-*d*_6_): δ 1.3 (brs, *W*_1/2_ = 325 Hz). MS calculated for C_36_H_28_Cl_2_Cu_2_OP_2_: 736.55, found: *m*/*z* 736.0 [M]^+^. Anal. Calcd. For C_36_H_28_Cl_2_Cu_2_OP_2_: C, 58.70; H, 3.83; N, 0.00. Found: C, 58.52; H, 3.96; N, 0.04.

### 2.5. Oxygen-Sensing Operation

The testing atmosphere was controlled by pure N_2_ and pure O_2_ flows which were mixed with desired ratio and then imported into a quartz chamber. Both N_2_ and O_2_ flows were controlled by flowmeters. Each sample was immobilized in the quartz chamber and kept for at least 5 s to achieve atmosphere balance. Steady emission spectra were recorded by the F7000 (Hitachi) spectrometer under luminescence mode (5 nm × 5 nm). Each measurement was repeated three times to get a mean value.

For oxygen-sensing pics, quartz plates were pre-cleaned. Then CuPBr-COF or CuPCl-COF was dispersed in CH_2_Cl_2_ (50 mg/mL), oxygen-sensing pics were prepared by spin-coating method (600 rpm).

## 3. Results and Discussion

### 3.1. Characterization on CuPX and CuPX-COF, X = Br or Cl

#### 3.1.1. Single Crystal Structure of CuPBr

**Figure 1:**
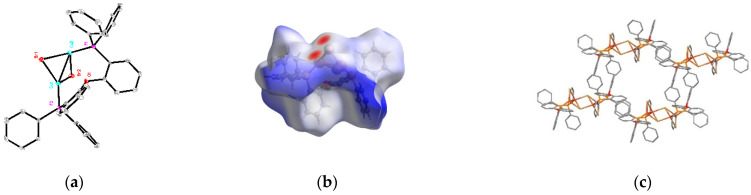
Single crystal structure of CuPBr (**a**), its Hirshfeld surface plotting (**b**) and its packing mode (**c**).

As depicted in Scheme 1 and Section 2.3, the dopant CuPX (X = Br or Cl) was in-situ synthesized in the micropores of Br-COF and Cl-COF. For comparison convenience, CuPX (X = Br and Cl) was synthesized as a reference compound under a similar condition, without Br-COF and Cl-COF. The molecular identity of CuPX (X = Br or Cl) has been confirmed above by NMR, MS and elemental analysis. Additionally, CuPBr single crystal was obtained and presented in Figure 1. Detailed geometric parameters are listed in Supplementary Materials. Two Cu(I) ions are coordinated by two Br^-^ ions and a POP ligand, with Cu…Cu distance of 2.62 Å which is rather close to the radius sum of two Cu atoms (1.28 + 1.28 Å). The two bridging Br^-^ ions help to stabilize these two Cu(I) ions. The crystal cell length values are measured as a = 12.36 Å, b = 13.80 Å and c= 13.98 Å. This small size ensures the successful loading of CuPBr in Br-COF micropores (with pore size ~2 nm), which will be further discussed below.

Owing to the free rotation of phenyl rings in POP ligand, each CuBr molecule is far away from the each other, with weak intermolecular interaction, as shown by its Hirshfeld surface plotting shown in Figure 1. Even in packing mode, there is no obvious aggregation or π-π interaction between CuPBr molecules. This is good news for an oxygen-sensing probe since the interaction between probe molecules always compromises sensing performance by barricading O_2_ impact, resulting in bi-exponential excited state lifetime and thus non-linear quenching behavior [9,10,16].

#### 3.1.2. Electronic Structure of CuPBr

**Figure 2:**
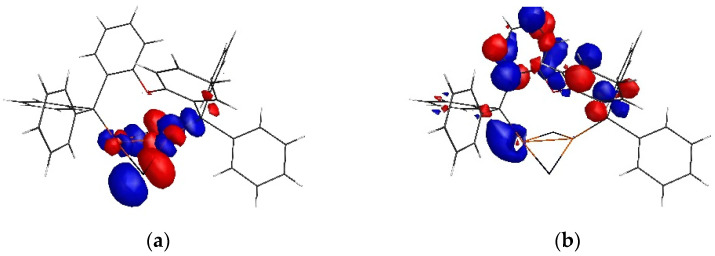
HOMO (**a**) and LUMO (**b**) plotting of CuPBr calculated at RB3LYP/LANL2DZ level, E_HOMO_ = −5.061 eV, E_LUMO_ = −1.045 eV.

It has been reported that an oxygen-sensing procedure based on luminescence quenching is generally a dynamic one, where ^3^O_2_ (ground state) attacks probe excited electrons, resulting in excited state ^1^O_2_ and probe emission quenching [9,10]. As a consequence, the electronic structure of probe plays an important role in controlling sensing sensitivity, response time and even the linearity of calibration curve. Most reported metal-based probes are charge-transfer-based (CT-based) ones [9,10,11,12,17]. The virtues of a CT-based probe include large Stokes shift to avoid excitation light interference, broad distribution of excited state electrons to increase collision probability with O_2_, and long lifetime to allow more collision chances with O_2_ [19]. The electronic structure of CuPBr is revealed by TD-DFT method [17,19]. It is observed from Figure 2 that the highest occupied molecular orbital (HOMO) of CuPBr is composed of Cu and Br atoms, with rather slim contribution from POP ligand, while its lowest unoccupied molecular orbital (LUMO) is basically the π* of the POP ligand, admixed with contribution from Cu d orbital. The onset electronic transition corresponds to a transition from HOMO to LUMO, with excitation energy of 3.40 eV. It is thus assigned as a mixed character of (M + X)LCT. Here M means metal, X means halogen atom, L denotes phosphorous ligand, and CT means, as above mentioned, charge transfer. The observation of such CT transition shall favor the oxygen-sensing behavior of CuPBr, which will be confirmed below. In addition, this excitation energy is found much higher than those of [Cu(N-N)(POP)]^+^ (<3.0 eV) [19]. We attribute this high transition energy to the strong coordination effect from Br^-^ ions.

#### 3.1.3. Simulated Structure of X-COF

**Figure 3:**
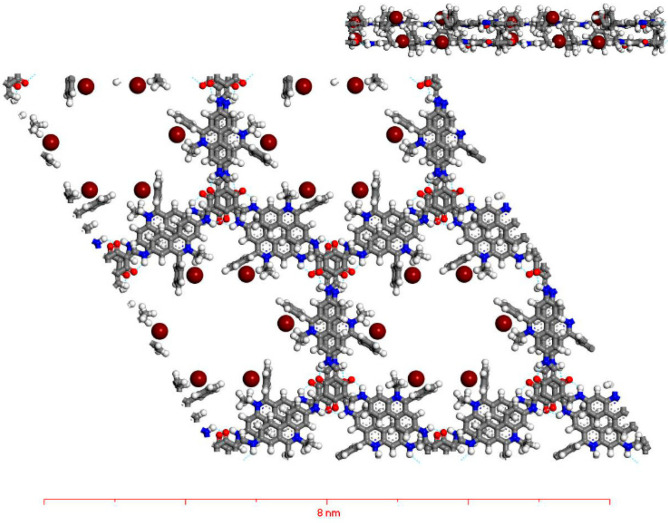
Top view of simulated Br-COF structure, inset: side view of offset ABA staking of Br-COF layers.

The above analysis on CuPBr single crystal has suggested that its molecular size is no larger than 1.5 nm. Aiming at a tentative evaluation on the possible CuPX doping in X-COF micropores, the monolayer structure and stacking structure of X-COF should be simulated. The monolayer structure of Br-COF and its energy-minimized stacking mode were optimized by universal force-field model and shown as Figure 3 [15]. The diameter of Br-COF micropore is measured as ~2.2 nm which is large enough to load CuPBr (a = 12.36 Å, b = 13.80 Å and c = 13.98 Å). There are three Br^-^ ions in each Br-COF micropore, two of them are able to react with [Cu(CH_3_CN)_2_(POP)]BF_4_, to form one CuPBr molecule. In other words, theoretically, there should be one and only one CuPBr molecule in each Br-COF micropore, due to the restriction of geometric space and charge balance. It is till observed from Figure 3 that Br-COF layers tend to take an offset ABA staking which is an energy-favored structure (285 kcal/mol), compared to the geometrical energy values of 532 kcal/mol for ideal AA stacking mode and 451 kcal/mol for ideal AB staking mode. Considering that Cl-COF was obtained with Br-COF as a starting compound by an ionic exchange reaction, they should have nearly identical backbone microstructure, except for their different counterions (Br^-^ for Br-COF and Cl^-^ for Cl-COF).

#### 3.1.4. XRD Analysis, SEM Morphology, IR Spectra and Microporous Structure

**Figure 4:**
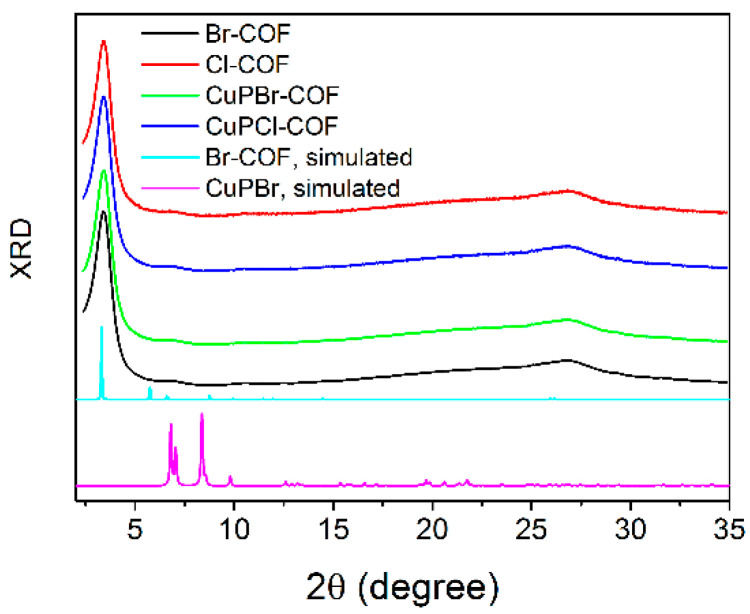
Recorded XRD curves of X-COF, CuPX-COF, along with simulated XRD peak of Br-COF and CuPBr, X = Br and Cl.

The recorded XRD curves of X-COF and CuPX-COF (X = Br and Cl) are shown in Figure 4. There is a sharp XRD peak around 3.3° and a broad one around 27° in Br-COF XRD curve. The first peak matches the simulated XRD peak of Br-COF. After ionic exchange and bridging-reaction with [Cu(CH_3_CN)_20_(POP)]BF_4_, these two peaks are well preserved in Cl-COF and CuPX-COF, with no obvious spectral shift or relative intensity variation. This observation suggests that the hexagonal microstructure has been constructed and well preserved after loading Cu-based probes. On the other hand, no detectable XRD peaks from dopant CuPBr are observed, which means that dopant molecules have been uniformly distributed into COF micropores, with no aggregation or phase separation (More explanation words can be found from Supplementary Materials).

**Figure 5:**
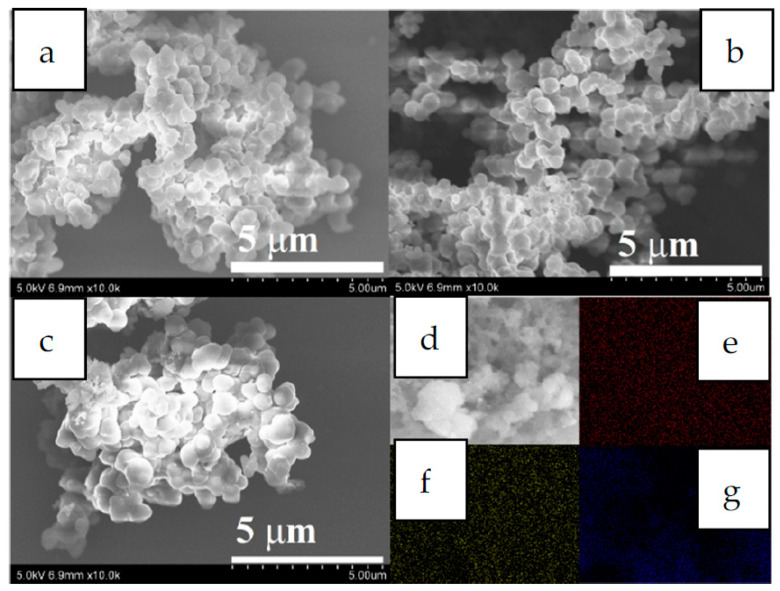
SEM mages of CuPBr-COF (**a**), CuPCl-COF (**b**) and Br-COF (**c**), and elemental mapping of CuPBr-COF ((**d**), SEM, (**e**), C, (**f**), N, (**g**), Cu).

To confirm the above statement, SEM images of Br-COF, CuPBr-COF and CuPCl-COF are shown in Figure 5. Spherical-liked nanoparticles with diameter of ~1 μm are observed for Br-COF. After ionic exchange and bridging-reaction with [Cu(CH_3_CN)_20_(POP)]BF_4_, the spherical morphology has been well preserved, admixed with some structural fragments. It seems that these gentle operations (ionic exchange and bridging-reaction at ambient condition) have slim impact on Br-COF structure. The elemental mapping of CuPBr-COF is shown in Figure 5 as well. Uniform distribution is observed for Cu element, with no obvious aggregation, suggesting that CuPBr molecules have been uniformly distributed in COF micropores (see Figure S3 of Supplementary Materials for more elemental mapping photos and TEM images).

**Figure 6:**
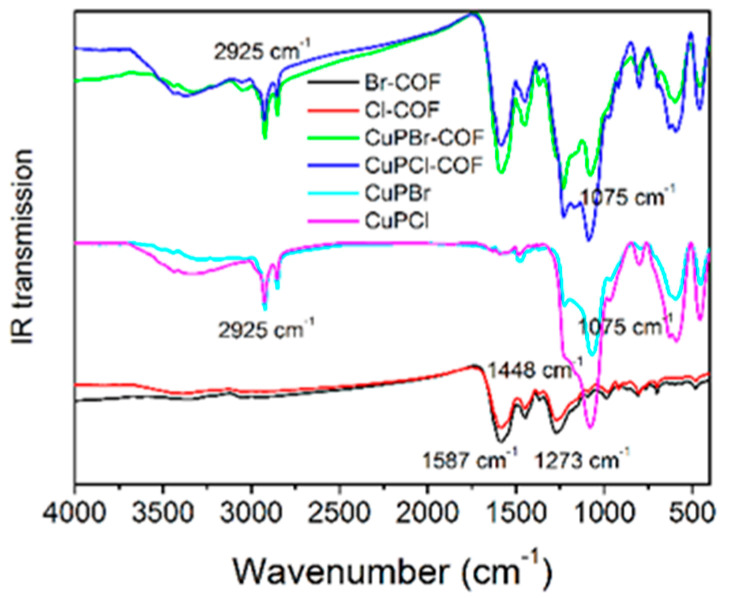
IR spectra of CuPX, CuPX-COF and X-COF, X = Br, Cl.

The successful dopant loading in CuPX-COF is further analyzed with IR spectral comparison between CuPX, CuPX-COF and X-COF, X = Br, Cl. The IR spectra of CuPX are similar to each other owing to their rather similar molecular composition. As shown in Figure 6, there are two characteristic bands, peaking at 2925 cm^−1^ and 1075 cm^−1^. The former peak is assigned as the IR absorption from Cu-X cluster, while the latter one is attributed to the off-plane bending vibration of C-H bond from POP ligand [20]. The IR spectra of Br-COF and Cl-COF are nearly identical to each other due to their identical COF structure, peaking at 1587 cm^−1^, 1448 cm^−1^ and 1273 cm^−1^, respectively. The first two IR bands are attributed to vibrations of C = C bonds of phenyl rings, while the latter one is considered as in-plane bending vibration of C-H bond [20]. All above mentioned IR peaks are traced from the IR spectra of CuPX-COF (X = Br and Cl), especially the IR peaks from Cu-X cluster (2925 cm^−1^). It is thus confirmed that dopant CuPX has been successfully in-situ synthesized in the micropores of X-COF, X = Br, Cl.

**Figure 7:**
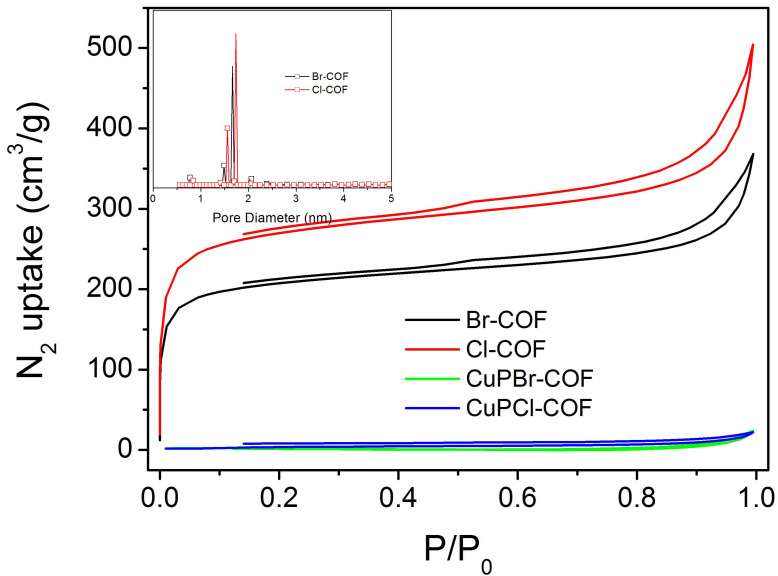
N_2_ adsorption/desorption isotherms of CuPX-COF and X-COF, X = Br, Cl. Inset: pore distribution of X-COF.

The above statement is finally confirmed by the N_2_ adsorption/desorption isotherms of CuPX-COF and X-COF, X = Br, Cl, as shown in Figure 7. As for X-COF, a sharp N_2_ uptake is observed at low pressure, suggesting the presence of micropores in X-COF samples. Their Brunauer–Emmett–Teller (BET) surface area values are determined as 775 m^2^/g for Br-COF and 955 m^2^/g for Cl-COF, with pore size values of 16.6 Å and 17.3 Å, respectively. The smaller porous parameters of Br-COF than those of Cl-COF are explained by the larger size of Br^-^ (3.92 Å) than Cl^-^ (3.62 Å). After in-situ dopant synthesis/loading, their BET surface area values are greatly decreased (lower than 10 m^2^/g). It is thus confirmed that CuPX dopant has been successfully in-situ synthesized/loaded into COF micropores.

#### 3.1.5. Doping Level Determined by Elemental Data and Thermal Analysis

The doping level of CuPX in CuPX-COF is then discussed by their elemental data and thermal gravimetric analysis (TGA) curves. As mentioned in Section 3.1.3, there are three halogen atoms in each X-COF micropore, two of them are able to react with [Cu(CH_3_CN)_2_(POP)]BF_4_, to form one CuPX molecule (X = Br and Cl). In other words, theoretically, there should be one and only one CuPX molecule in each X-COF micropore, due to the restriction of geometric space and charge balance. The recorded C/N/H composition of CuPX-COF is comparable to the theoretical C/N/H composition of CuPX-COF, which confirms the 1:1 loading in each X-COF micropore.

**Figure 8:**
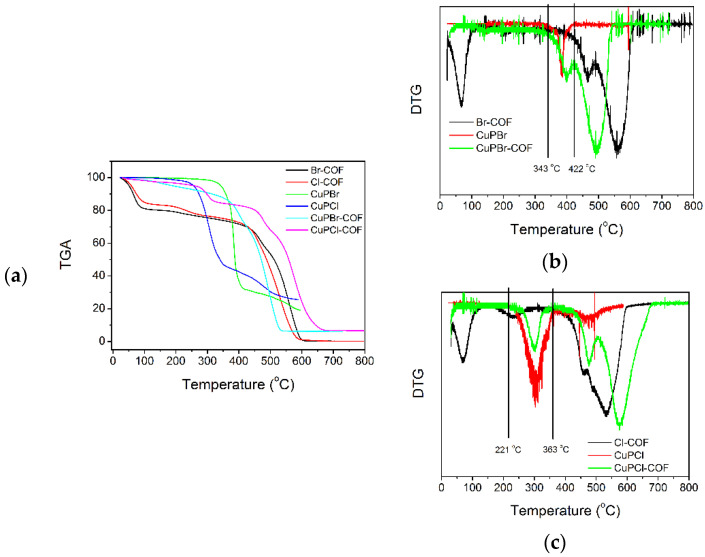
TGA (**a**) and DTG (**b**,**c**) curves of of CuPX, CuPX-COF and X-COF, X = Br, Cl.

A more precise result is given via the TGA curves of X-COF, CuPX and CuPX-COF, as shown in Figure 8. To assist weight loss assignment, differential thermal gravimetric (DTG) curves are plotted. Br-COF and Cl-COF have three endothermic peaks, centering at 68 °C, 466 °C and 560 °C. The former one is attributed to the thermal release of adsorbent molecules such as water, while the latter two ones are attributed to the thermal decomposition and collapse of COF structure. CuPBr and CuPCl depict mono endothermic peak, centering at 384 °C and 305 °C, respectively. The endothermic peaks of CuPX-COF are composed of those from dopant CuPX and host X-COF, with minor temperature shift, due to the interaction between CuPX and X-COF. The weight loss values of CuPX are determined as 63.6% (343–422 °C) for CuPBr and 52.5% (221–363 °C) for CuPCl, respectively. While, corresponding weight loss values of CuPX-COF within the same temperature region are determined as 17.2% for CuPBr-COF and 12.1% for CuPCl-COF. The doping levels are thus calculated as 27.0% in CuPBr-COF and 23.1% for CuPCl-COF. These values are found rather close to their theoretical values, 22.6% for CuPBr-COF (C_162_H_120_N_18_O_12_Br_4_:C_36_H_28_Br_2_Cu_2_OP_2_) and 21.7% for CuPCl-COF (C_162_H_120_N_18_O_12_Cl_4_:C_36_H_28_Cl_2_Cu_2_OP_2_). It is thus confirmed that CuPX was loaded in X-COF micropores with a ratio of 1:1.

### 3.2. Photophysical Parameters of CuPX under N_2_ and O_2_: Quantum Yield and Lifetime

**Figure 9:**
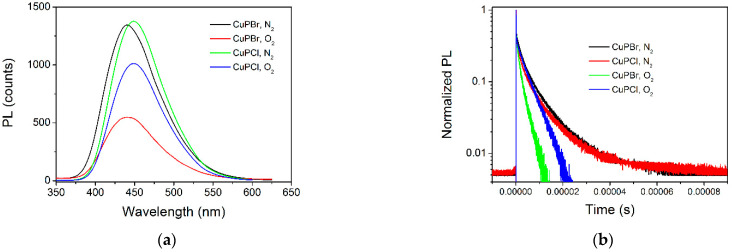
Emission spectra (**a**) of CuPX with excitation wavelength of 350 nm under pure N_2_ and pure O_2_. Emission decay dynamics of (**b**) of CuPX with excitation wavelength of 355 nm under pure N_2_ and pure O_2_, X = Br, Cl.

Some crucial photophysical parameters of CuPX are recorded so that their oxygen-sensing performance can be tentatively evaluated. It is observed from Figure 9 that, under pure N_2_ atmosphere, CuPX exhibits Gaussian-liked blue emission, peaking at 440 nm for CuPBr and 450 nm for CuPCl. Their emission quantum yields (Φ) are determined as 0.30 and 0.31, with excited state lifetime (τ) as long as 6.7 μs and 6.8 μs, respectively. These long-lived excited states suggest that they have a phosphorescent nature, which allows enough chances to be quenched by O_2_. Given pure O_2_ atmosphere, CuPX emission is obviously quenched, with emission quantum yields decreased to 0.11 for CuPBr and 0.24 for CuPCl, respectively. Their lifetimes are quenched to 2.5 μs and 5.2 μs. This observation suggests that CuPX emission is quenchable by O_2_, which endows CuPX with oxygen-sensing possibility. On the other hand, there is no obvious spectral shift or bandshape change, indicating that the CT-based excited state is well preserved. The absorption spectra of CuPBr upon pure N_2_ and pure O_2_ atmospheres are recorded and compared in Figure S4 (Supplementary Materials). No obvious difference is observed. This is because the oxygen-sensing mechanism is a dynamic one, via a dynamic collision between CuPBr triplet excited state and O_2_ molecules. CuPBr ground state takes no participation in the sensing procedure. As a consequence, the electronic transition of CuPBr ground state (namely its absorption) is immune from O_2_ level variation. Aiming at a better understanding on CuPX excited state, corresponding emissive and non-emissive probabilities (k_r_ and k_nr_) are calculated by Equations (1) and (2).

Φ = k_r_/(k_r_ + k_nr_) (1)

τ = 1/(k_r_ + k_nr_) (2)

The k_r_ and k_nr_ of CuPBr are calculated as 4.4 × 10^4^ s^−1^ and 10.3 × 10^4^ s^−1^ under pure N_2_ atmosphere, 4.3 × 10^4^ s^−1^ and 35.1 × 10^4^ s^−1^ under pure O_2_ atmosphere. The non-emissive probability is increased by 3-fold. A similar observation is observed for CuPCl. Its k_r_ and k_nr_ values are calculated as 4.5 × 10^4^ s^−1^ and 10.1 × 10^4^ s^−1^ under pure N_2_ atmosphere, 4.5 × 10^4^ s^−1^ and 14.4 × 10^4^ s^−1^ under pure O_2_ atmosphere. This observation confirms the oxygen-sensing possibility of CuPX. A schematic presentation for the CuPX phosphorescence and corresponding sensing mechanism is shown as Figure S5 (Supplementary Materials). But the k_nr_ value of CuPCl is increased by only 1.5-fold under pure O_2_ atmosphere, compared to that under pure N_2_ atmosphere.

### 3.3. Oxygen-Sensing Performance of CuPX-COF

#### 3.3.1. Emission Spectra under Various O_2_ levels

**Figure 10:**
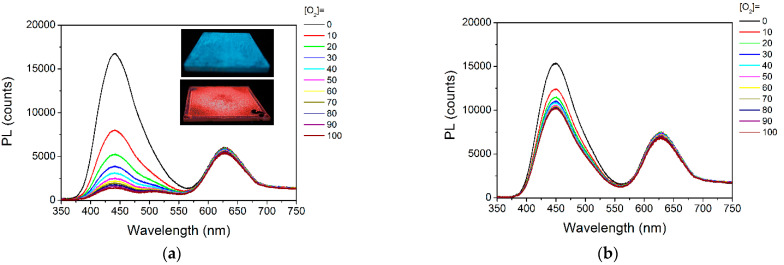
Emission spectra of CuPBr-COF (**a**) and CuPCl-COF (**b**) upon various O_2_ levels. Inset: photos of CuPBr-COF upon pure N_2_ (top inset) and pure O_2_ (bottom inset) atmosphere upon excitation light of 350 nm.

The oxygen-sensing performance of CuPX-COF (X = Br and Cl) is tentatively discussed by comparing its steady emission spectra upon addition of various O_2_ levels. It is observed from Figure 10 that CuPX-COF exhibits characteristic emission bands from CuPX and X-COF, peaking at 440 nm and 630 nm for CuPBr-COF, 450 nm and 630 nm for CuPCl-COF, respectively. The former emission band of each CuPX-COF sample comes from dopant CuPX, while the latter one comes from host X-COF. It is observed that the dopant blue emission is obviously quenched by increasing O_2_ level, but X-COF red emission is just slightly quenched. In this case, an emission color change from blue (under pure N_2_) to red (under pure O_2_) is observed, as shown in Figure 10. To reveal the nature of COF red emission quenching at 630 nm (O_2_ quenching or photodegradation), emission monitoring of CuPBr-COF at 630 nm upon pure N_2_ ad pure O_2_ atmospheres is performed and shown as Figure S6 (Supplementary Materials). Upon pure O_2_-pure N_2_-pure O_2_ cycles, COF red emission is correspondingly quenched–recovered–quenched. As a consequence, we tentatively conclude that the COF emission quenching is mainly caused by O_2_ quenching effect, instead of photodegradation.

For a comparison between CuPBr-COF and CuPCl-COF, sensitivity is defined as I_0_/I_100_, where I_0_ means the emission intensity at 0% O_2_ and I_100_ denotes that at 100% O_2_, respectively. The sensitivity values of CuPBr-COF and CuPCl-COF are determined as 12.25 and 1.50, respectively, where CuPBr-COF shows a much higher sensitivity than CuPCl-COF. Considering their nearly identical geometric structure and composition, we attribute this sensitivity difference to the heavy-atom-turbulence effect of Br in CuPBr, which increases the phosphorescent nature of CuPBr emission, favoring ^3^O_2_ attack. CuPCl emission has less phosphorescent composition to be quenched by ^3^O_2_, leading to its limited sensitivity. This observation explains why CuPCl excited state (τ = 6.8 μs in pure N_2_ vs. τ = 5.2 μs in pure O_2_) is less quenched in pure O_2_, compared to the case of CuPBr (τ = 6.7 μs in pure N_2_ vs. τ = 2.5 μs in pure O_2_). In addition, the porous structure of X-COF offers a high specific-surface-to-volume ratio, which improves sensitivity by allowing more dopant molecules to meet and be quenched by O_2_.

The selectivity of CuPBr-COF is tentatively discussed via its emission spectra upon various gases, including CO_2_, H_2_, CH_4_, C_2_H_2_ and moisture. It is observed from Figure S7 (Supplementary Materials) that CuPBr-COF emission bands (440 nm and 630 nm) are nearly constant upon the first four gases, indicating a good selectivity. This is because they are closed-shell structure and are not able to accept energy from CuPBr phosphorescence. Moisture, however, has quenching effect on CuPBr emission since H_2_O may quench the triplet CuPBr excited state. Thus, to ensure precise and reliable result, testing gas should be dried before sensing.

#### 3.3.2. Response and Recovery

**Figure 11:**
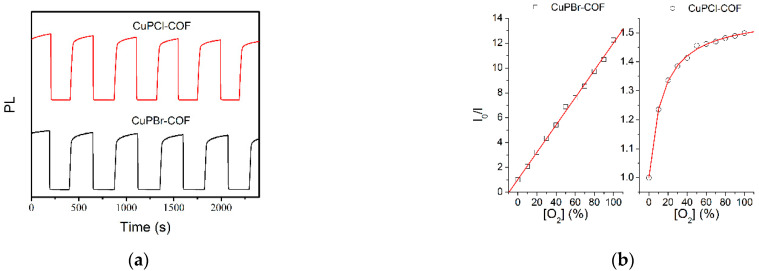
Emission monitoring (**a**) and Stern-Volmer plots (**b**) of CuPX-COF, X = Br, Cl.

Aiming at an evaluation on the correlation between O_2_ presence and CuPX-COF emission, CuPX-COF emission is monitored when testing atmosphere is switched between pure N_2_ and pure O_2_. It is observed from Figure 11 that CuPBr-COF (440 nm) and CuPCl-COF (450 nm) emission remains at a high level in pure N_2_ atmosphere. Upon pure O_2_ atmosphere, their emission is instantly quenched to a low level and preserved. Their emission intensity can be recovered back to a high level given a pure N_2_ atmosphere. To compare their sensing response performance, response time is defined as the time for each sample to lose 95% of its initial emission intensity (from pure N_2_ to pure O_2_), while recovery time is defined as the time for each sample to recover 95% of its initial emission intensity (from pure O_2_ to pure N_2_). The response time values of CuPBr-COF and CuPCl-COF are determined as 13 s and 13 s, while their recovery time values are determined as 38 s and 40 s. Their rather similar response/recovery performance is attributed to their nearly identical geometric structure and composition. The recovery time is 3-fold longer than the response time. This is because the recovery process is a dynamic diffusion-controlled one, [21]. In addition, it is observed that there is a gradual smooth increase for CuPX-COF emission in pure N_2_ atmosphere, indicating the adsbrobed/residual O_2_ in sample, which is attributed to the micropores of X-COF having high affinity for O_2_ gas.

#### 3.3.3. Calibration Curve

The above discussion has confirmed a dynamic quenching mechanism of CuPX-COF for O_2_. In this case, the steady emission intensity upon various oxygen levels can be analyzed by Stern–Volmer equation described by Equation (3) [22,23]. Here, K_sv_ is Stern-Volmer constant, [O_2_] means oxygen level.

I_0_/I = C + K_sv_[O_2_] (3)

An ideal Stern–Volmer equation should be a linear one, given a condition that probe molecules are uniformly distributed and their emission is homogeneously quenched by O_2_. The Stern-Volmer plots of CuPBr-COF follow a linear response, as expected, with fitting equation of I_0_/I = 1.033 + 0.110*[O_2_], R^2^ = 0.999. But those of CuPCl-COF are non-linear ones and fail to obey Equation (3). It has been above mentioned that CuPCl emission has less phosphorescent composition than CuPBr emission. As a consequence, it is assumed that there should be multiple sensing sites in CuPCl-COF, some of them are oxygen-quenchable, while the others are not. In this case, a two-site Demas model should be applied to describe CuPCl-COF steady emission spectra, as shown by Equation (4) [22,23]. Here, f_1_ and f_2_ are fractional contributions of sensing sites (f_1_ + f_2_ = 1), K_sv1_ and K_sv2_ are corresponding Stern-Volmer constants of sensing sites.

I_0_/I = 1/{f_1_/(1 + K_sv1_[O_2_]) + f_2_/(1 + K_sv2_[O_2_])} (4)

Corresponding fitting equation is obtained as I_0_/I = 1/{0.362/(1 + 0.112[O_2_]) + 0.638/(1 + 0.00001[O_2_])}, R^2^ = 0.999. It is observed that K_sv1_ is comparable to that of CuPBr-COF, but K_sv2_ is close to 0, which means that its emission is nearly non-quenchable by O_2_. This conclusion is consistent with the obvious emission of CuPCl under pure O_2_ atmosphere. Some important sensing parameters of CuPX-COF are compared to literature ones in Table 1. It is observed that CuPBr-COF is a promising one, showing virtues of high sensitivity, linear calibration curve, short response time, along with visual color change during sensing procedure.

## 4. Conclusions

As a conclusion, this paper reported two oxygen probes based on halogen-bridged Cu cluster and their oxygen-sensing performance. They were loaded into COF micropores by an in-situ method. The resulting composite samples were characterized in detail to confirm the successful probe loading, including single crystal analysis, DFT calculation, XRD, SEM, IR, N_2_ adosprtion/desorption, and TGA. The doping level was determined as ~22%. The halogen-bridged Cu clusters showed blue emission peaking at ~440 nm, while COF host showed red emission peaking at 630 nm. These halogen-bridged Cu clusters had long emissive lifetime of ~6.7 μs and high emission quantum yield of 0.30 in pure N_2_ atmosphere. Given pure O_2_ atmosphere, lifetime and quantum yield were quenched to 2.5 μs and 0.11, showing oxygen-sensing possibility. A linear oxygen-sensing calibration curve was observed, with sensitivity of 12.25, response time of 13 and recovery time of 38 s. Sample emission color was changed from blue to red when testing atmosphere was changed from pure N_2_ to pure O_2_, which was detectable by eyes. It was found that Br-containing probe was superior to Cl-containing one by showing higher sensitivity and linear calibration curve, due to the heavy atom turbulence effect. For further effort, sensitivity can be further improved by incorporating more heavy atoms into probe structure. This work proposed a method of constructing an oxygen-sensing system by in-situ (one-step) synthesizing light-emitting Cu(I) cluster in luminescent porous COF, so that probe molecules can be uniformly distributed in COF micopores. Sensitivity and linearity of the calibration curve can be improved by this method, compared to the sensing systems prepared by two-step methods.

## Figures and Tables

**Table 1:** Key sensing parameters of CuPX-COF and literatures.

System	I_0_/I_100_	K_sv_ (O_2_%^−1^)	T_res_ (s)	T_rec_ (s)	Linearity	Citation
CuPBr-COF	12.25	0.110	13	38	linear	this
CuPCL-COF	1.50	0.112/0.00001	13	40	no	this
[Cu(POP)(PTZ)]BF_4_-MCM-41	11.16	0.500/0.002	4	78	no	[24]
[Cu(dpephos)(pip)]BF_4_-SBA-15	7.44	2.043/0.002	3	178	no	[25]
[Cu(dpephos)(epip)]BF_4_-SBA-15	5.68	1.508/0.002	4	158	no	[25]
[Cu(dpephos)(nip)]BF_4_-SBA-15	5.51	1.816/0.001	3	119	no	[25]
[Cu(dpephos)(enip)]BF_4_-MCM-41	5.95	0.843/0.008	8	39	no	[25]

## Data Availability

Detailed geometric parameters of CuPBr are listed in Supplementary Materials.

## Supplementary Materials

The following are available online at https://www.mdpi.com/article/10.3390/ma15134525/s1, Figures S1 and S2: ^1^H NMR of CuPBr and CuPCl, Figure S3: Elemental mapping of CuPBr-COF, Figure S4: Absorption spectra of CuPBr film under pure N_2_ and pure O_2_ atmospheres, Figure S5: A schematic presentation for the CuPX phosphorescence and corresponding sensing mechanism, Figure S6: Emission monitoring of CuPBr-COF at 630 nm upon pure N_2_ ad pure O_2_ atmospheres, Figure S7: Emission spectra of CuPBr-COF upon various gases, including CO_2_, H_2_, CH_4_, C_2_H_2_ and moisture, Detailed geometric parameters of CuPBr.
