# Supplementary material for: Eye-Visible Oxygen Sensing via In-Situ Synthesizing Blue-Emitting Cu(I) Cluster in Red-Emitting COF: Characterization and Performance
Source: Materials (Basel). 2022 Jun 27;15(13):4525. doi: 10.3390/ma15134525 (PMC9267314; doi:10.3390/ma15134525)
Supplement: Supplementary file 1 [file materials-15-04525-s001.zip › materials-1756013-supplementary.pdf]

materials

MDPI

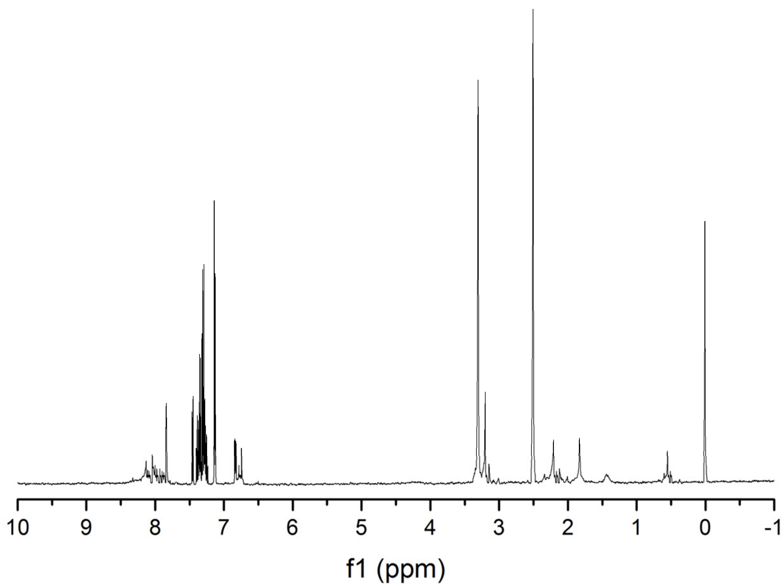

Figure S1. ^{1}H NMR of CuPBr.

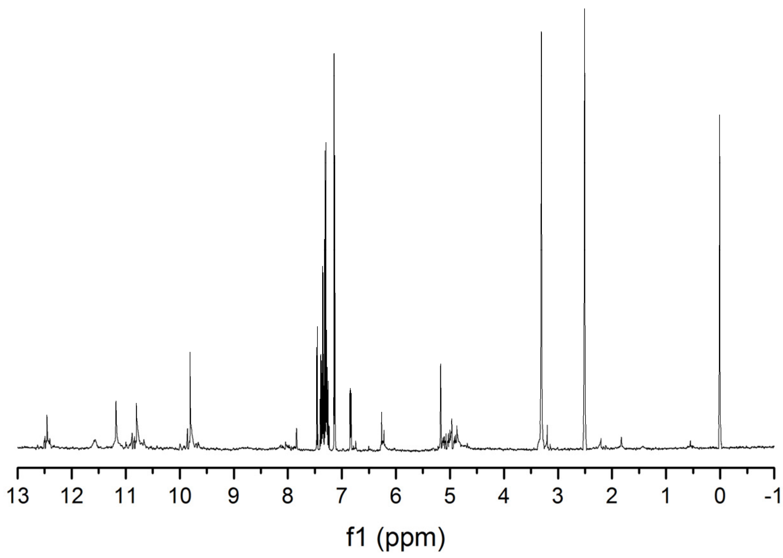

Figure S2. ^{1}H NMR of CuPCl.

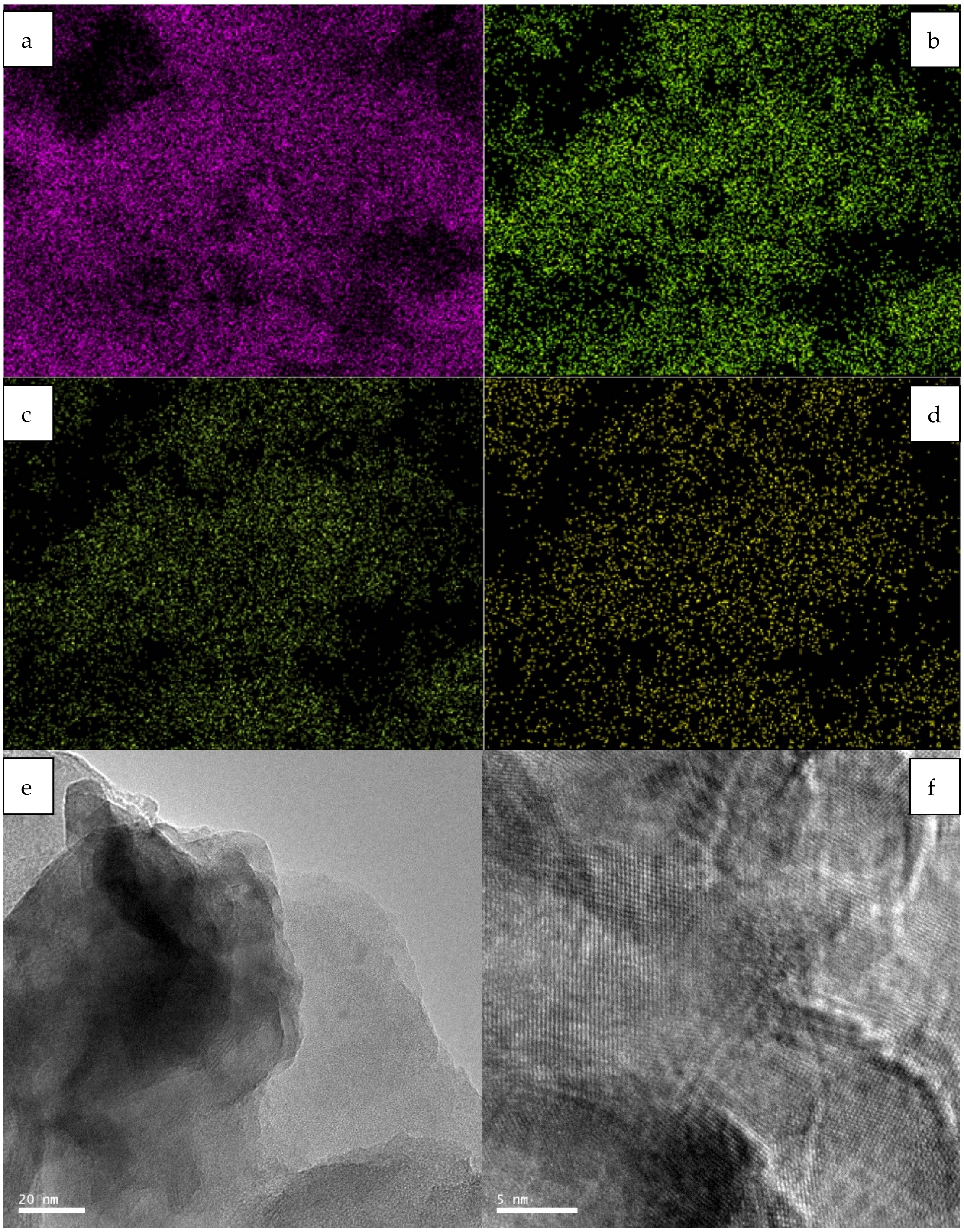

Figure S3. Elemental mapping of CuPBr-COF, a, C; b, O; c, Cu; d, P. TEM images of CuPBr, e and f.

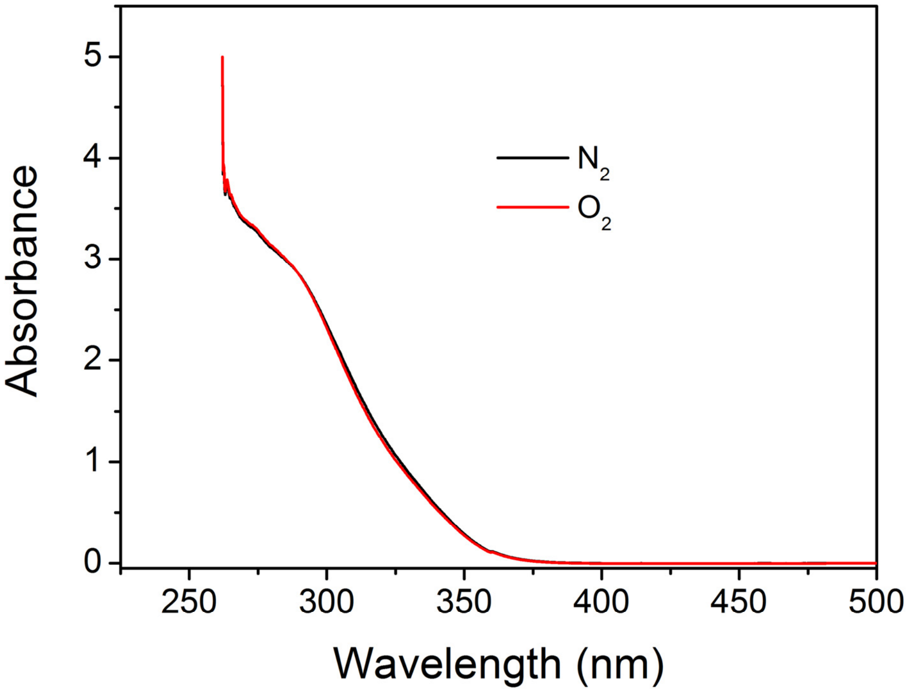

Figure S4. Absorption spectra of CuPBr film under pure N_2 and pure O_2 atmospheres.

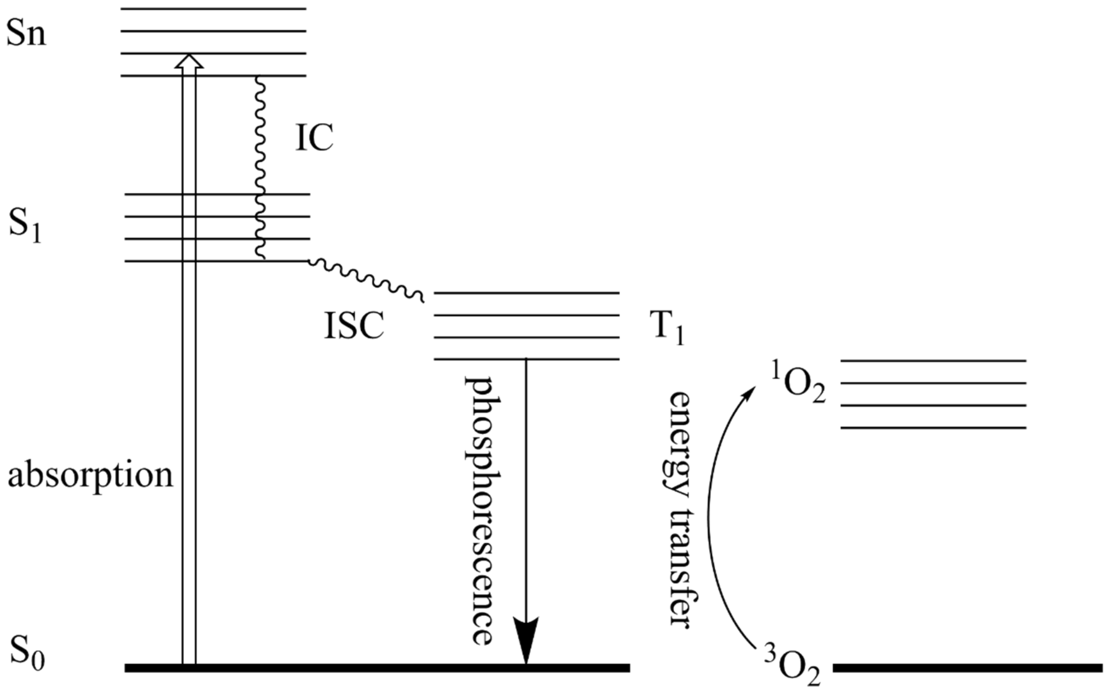

Figure S5. A schematic presentation for the CuPX phosphorescence and corresponding sensing mechanism. S_n means singlet excited state, S_0 is ground state, S_1 is the first singlet excited state, T_1 is the first triplet state, IC means internal conversion, ISC means intersystem conversion.

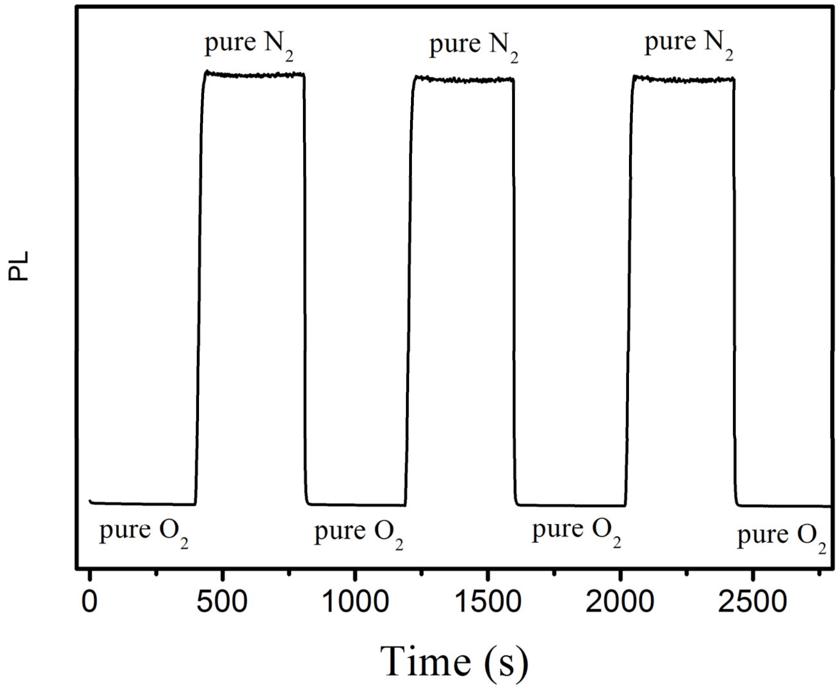

Figure S6. Emission monitoring of CuPBr-COF at 630 nm upon pure N_2 ad pure O_2 atmospheres.

To reveal the nature of COF red emission quenching at 630 nm (O_2 quenching or photodegradation), emission monitoring of CuPBr-COF at 630 nm upon pure N_2 ad pure O_2 atmospheres is performed and shown as Figure S3 (Supporting Information). Upon pure O_2-pure N_2-pure O_2 cycles, COF red emission is correspondingly quenched-recovered-quenched. As a consequence, we tentatively conclude that the COF emission quenching is mainly caused by O_2 quenching effect, instead of photodegradation.

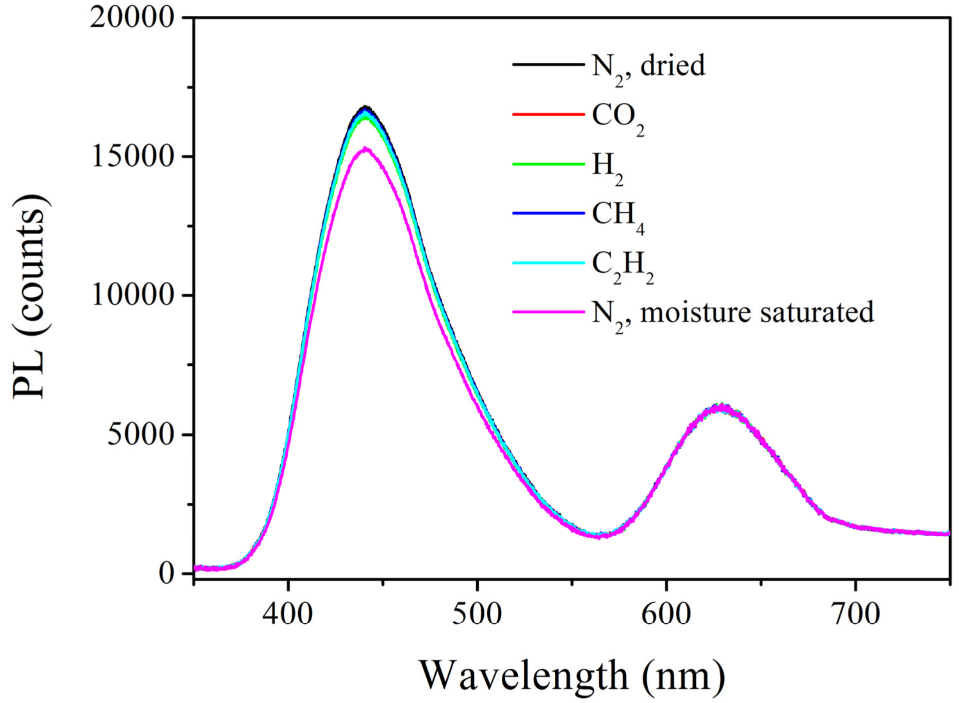

Figure S7. Emission spectra of CuPBr-COF upon various gases, including CO_2, H_2, CH_4, C_2H_2 and moisture, excitation wavelength = 350 nm.

Explanation about PXRD curves

We used FullPro 2016 (PCR Editor) to do Rietveld refinement on these PXRD curves, but no obvious improvement was obtained. We are giving an explanation as follows.

As shown in Figure 4, the PXRD curves of CuPX-COF are nearly identical to those of X-COF. No XRD peaks from CuPX are observed since CuPX is uniformly distributed in COF micropores, instead of being aggregated as nanocrystals. The PXRD curve of X-COF shows one dominant peak (at 3.3 °), no other obvious peaks are detected. This observation is consistent with the simulated XRD pattern of Br-COF. In addition, it should be mentioned that the crystalinity of COF materials was limited (not as good as inorganic nanocrystals), resulting in poor and low XRD reflections/signals. The above two reasons shall be responsible for the unsatisfactory PXRD curves.

Detailed geometric parameters of CuPBr

```
data_CuPBr
_symmetry_cell_setting             triclinic
_symmetry_space_group_name_H-M     'P -1'
_symmetry_Int_Tables_number        2
_space_group_name_Hall             '-P 1'
loop_
_symmetry_equiv_pos_site_id
_symmetry_equiv_pos_as_xyz
1 x,y,z
2 -x,-y,-z
_cell_length_a                     12.3611(14)
_cell_length_b                     13.7965(15)
_cell_length_c                     13.9785(16)
```

_cell_angle_alpha 81.085(2)
_cell_angle_beta 68.241(2)
_cell_angle_gamma 65.282(2)
_cell_volume 2011.19
loop_
_atom_site_label
_atom_site_type_symbol
_atom_site_fract_x
_atom_site_fract_y
_atom_site_fract_z
C1 C -0.004367 0.286474 0.264874
C2 C 0.046084 0.176662 0.261149
H2 H 0.133492 0.140161 0.234198
C3 C -0.030361 0.119209 0.296637
H3 H 0.005809 0.045171 0.291811
C4 C -0.1585 0.171232 0.338588
H4 H -0.209838 0.132823 0.363394
C5 C -0.211413 0.280833 0.344015
H5 H -0.298876 0.316786 0.371368
C6 C -0.135016 0.337306 0.309
H6 H -0.171722 0.411251 0.31502
C7 C 0.239113 0.277672 0.129361
C8 C 0.256984 0.265704 0.027702
H8 H 0.191072 0.305915 0.002817
C9 C 0.368569 0.196568 -0.038161
H9 H 0.377941 0.190654 -0.106425
C11 C 0.121126 0.394455 0.318221
C12 C 0.174707 0.468352 0.307881
C13 C 0.20143 0.490249 0.387559
H13 H 0.236856 0.540045 0.378896
C14 C 0.174979 0.437271 0.480966
H14 H 0.193261 0.450867 0.535093
C16 C 0.306131 0.541432 0.172397
C17 C 0.294015 0.642936 0.133483
C18 C 0.403657 0.662498 0.087949
H18 H 0.398439 0.729066 0.05914
C19 C 0.520053 0.584855 0.084773
H19 H 0.592305 0.599828 0.055521
C20 C 0.529554 0.485078 0.124865
H20 H 0.608166 0.432533 0.122424
C21 C 0.121944 0.36513 0.492538
H21 H 0.104067 0.329731 0.555008
C22 C 0.166189 0.850001 0.054381
C23 C 0.182802 0.932363 0.08409
H23 H 0.184133 0.932658 0.150128
C24 C 0.421663 0.463279 0.168815
H24 H 0.427518 0.395978 0.195767
C25 C 0.09465 0.344108 0.413373
H25 H 0.057677 0.295195 0.423437
C40 C 0.197593 1.01503 0.016203
H40 H 0.20889 1.06994 0.037002

C41 C 0.195477 1.0153 -0.081011
H41 H 0.204829 1.07067 -0.12611
C42 C 0.179631 0.934283 -0.112002
H42 H 0.179061 0.934206 -0.178332
C43 C 0.164339 0.851833 -0.044054
H43 H 0.152739 0.797346 -0.065309
C44 C 0.466492 0.136059 -0.001572
H44 H 0.542487 0.088833 -0.045147
C45 C 0.451396 0.145783 0.099235
H45 H 0.517858 0.105348 0.123438
C46 C 0.07614 0.800511 0.270143
C47 C 0.153021 0.795545 0.323928
H47 H 0.240511 0.761496 0.294396
C48 C -0.054167 0.849091 0.317843
H48 H -0.107218 0.851818 0.283708
C49 C 0.340431 0.21383 0.164342
H49 H 0.331455 0.21819 0.232788
C50 C 0.101608 0.840175 0.41996
H50 H 0.153834 0.838139 0.454625
C51 C -0.027352 0.887752 0.464281
H51 H -0.062221 0.917085 0.52981
C52 C -0.106234 0.893022 0.413918
H52 H -0.193682 0.925945 0.444653
O1 O 0.195745 0.521585 0.21414
P1 P 0.086551 0.369417 0.210149
P2 P 0.137226 0.743926 0.141821
CU1 Cu 0.020447 0.675076 0.115083
CU2 Cu -0.018051 0.500343 0.124429
BR1 Br 0.121553 0.5657 -0.040222
BR2 Br -0.177909 0.669097 0.217237
